# Carnitine Palmitoyltransferase 1 Regulates Prostate Cancer Growth under Hypoxia

**DOI:** 10.3390/cancers13246302

**Published:** 2021-12-15

**Authors:** Leslimar Rios-Colon, Pawan Kumar, Susy Kim, Mitu Sharma, Yixin Su, Ashish Kumar, Sangeeta Singh, Nalexus Stocks, Liang Liu, Molishree Joshi, Isabel R. Schlaepfer, Deepak Kumar, Gagan Deep

**Affiliations:** 1 Department of Cancer Biology, Wake Forest Baptist Medical Center, Winston-Salem, NC 27157, USA; lrioscol@wakehealth.edu (L.R.-C.); chauhan2k1@gmail.com (P.K.); sukim@wakehealth.edu (S.K.); misharma@wakehealth.edu (M.S.); ysu@wakehealth.edu (Y.S.); ashish.kumar@wakehealth.edu (A.K.); sasingh@wakehealth.edu (S.S.); nalexusstocks@gmail.com (N.S.); liang.Liu@wakehealth.edu (L.L.); 2 Julius L. Chambers Biomedical Biotechnology Research Institute, North Carolina Central University, Durham, NC 27707, USA; dkumar@nccu.edu; 3 Division of Pathology, ICAR—Indian Veterinary Research Institute, Izatnagar 243122, India; 4 Center for Cancer Genomics and Precision Oncology, Wake Forest Baptist Medical Center, Winston-Salem, NC 27157, USA; 5 Wake Forest Baptist Comprehensive Cancer Center, Wake Forest Baptist Medical Center, Winston-Salem, NC 27157, USA; 6 Functional Genomics Facility, University of Colorado Anschutz Medical Campus, Aurora, CO 80045, USA; MOLISHREE.JOSHI@CUANSCHUTZ.EDU; 7 Division of Medical Oncology, University of Colorado Anschutz Medical Center, Aurora, CO 80045, USA; ISABEL.SCHLAEPFER@CUANSCHUTZ.EDU

**Keywords:** prostate cancer, hypoxia, carnitine palmitoyltransferase, sphere, xenograft

## Abstract

**Simple Summary:**

Cancer cell survival in hypoxia areas, with low oxygen and food supply as well as abundant waste material, is critical to their aggressiveness and associated with disease relapse and mortality. Therefore, it is vital to understand the molecular regulators of cancer cell survival under these harsh physiological conditions. In the present study, we assessed the role of a mitochondrial protein carnitine palmitoyltransferase (CPT1A) in regulating prostate cancer (PCa) cell survival and proliferation under hypoxic conditions in both cell culture and animal models. The results showed that CPT1A expression in PCa cells is key to their survival and proliferation in the hypoxic tumor microenvironment. These results have high translational significance in improving cancer prognosis and therapy.

**Abstract:**

Hypoxia and hypoxia-related biomarkers are the major determinants of prostate cancer (PCa) aggressiveness. Therefore, a better understanding of molecular players involved in PCa cell survival under hypoxia could offer novel therapeutic targets. We previously reported a central role of mitochondrial protein carnitine palmitoyltransferase (CPT1A) in PCa progression, but its role in regulating PCa survival under hypoxia remains unknown. Here, we employed PCa cells (22Rv1 and MDA-PCa-2b) with knockdown or overexpression of CPT1A and assessed their survival under hypoxia, both in cell culture and in vivo models. The results showed that CPT1A knockdown in PCa cells significantly reduced their viability, clonogenicity, and sphere formation under hypoxia, while its overexpression increased their proliferation, clonogenicity, and sphere formation. In nude mice, 22Rv1 xenografts with CPT1A knockdown grew significantly slower compared to vector control cells (~59% reduction in tumor volume at day 29). On the contrary, CPT1A-overexpressing 22Rv1 xenografts showed higher tumor growth compared to vector control cells (~58% higher tumor volume at day 40). Pathological analyses revealed lesser necrotic areas in CPT1A knockdown tumors and higher necrotic areas in CPT1A overexpressing tumors. Immunofluorescence analysis of tumors showed that CPT1A knockdown strongly compromised the hypoxic areas (pimonidazole+), while CPT1A overexpression resulted in more hypoxia areas with strong expression of proliferation biomarkers (Ki67 and cyclin D1). Finally, IHC analysis of tumors revealed a significant decrease in VEGF or VEGF-D expression but without significant changes in biomarkers associated with microvessel density. These results suggest that CPT1A regulates PCa survival in hypoxic conditions and might contribute to their aggressiveness.

## 1. Introduction

Prostate cancer (PCa) is the most common non-cutaneous cancer in men—accounting for more than 1 in 5 new diagnoses in men—with 248,538 new cases and more than 34,130 deaths estimated in the United States in 2021 [1]. Furthermore, PCa is the most commonly diagnosed cancer in African American (AA) men, accounting for nearly one-third of cancers diagnosed in this population and characterized by poorer PCa-specific outcomes in this population compared to Caucasian American (CA) men [2]. PCa mortality is mainly driven by metastatic castration-resistant prostate cancer (mCRPC), which is defined by progression to metastatic disease coupled with a developed resistance to current therapeutic alternatives [3,4,5]. It is essential to understand the mechanism driving the development of aggressive disease in order to develop innovative and effective treatment options that increase overall survival rates and improve the quality of life for patients.

A chronic condition of low oxygen in the tumor microenvironment, known as hypoxia, is an early event occurring in solid neoplasms that contributes to tumor aggressiveness. Cancer cells within this stressed microenvironment adopt many tumor-promoting characteristics, such as genomic instability, invasive behavior, and altered cellular bioenergetics [6]. Evidence suggests that the expression of hypoxia-related biomarkers is associated with poorer prognosis due to therapy failure and disease relapse in patients [6]. Furthermore, hypoxia has been demonstrated to be a predictor of poor biochemical outcomes [6,7,8,9,10]. Due to the important role of hypoxia in the tumor microenvironment, several approaches have been developed to target hypoxia-induced signaling and hypoxia inducible factor 1α (HIF-1α) inhibitors to circumvent therapeutic resistance [11,12]. Therefore, targeting hypoxia-induced signaling in tumors, such as inhibitors of HIF-1α and HIF-2α, may improve the efficacy of therapeutic regimens. However, since hypoxic areas of the tumor have decreased blood supply, further studies are needed to efficiently deliver these pharmacological agents to target hypoxic areas.

Cancer cells undergo complex metabolic reprogramming that includes increased lipid uptake, storage, and β-oxidation in order to compensate for the tremendous energetic demands created under microenvironmental stress, such as low-oxygen conditions [6,13,14,15,16]. For example, carnitine palmitoyltransferase 1 (CPT1A), the main regulator of β-oxidation located at the outer mitochondrial membrane, catalyzes the transport of long-chain fatty acids into the mitochondria, resulting in NAPDH and ATP production [17,18]. Studies have indicated that higher expression or activity of CPT1A plays a role in PCa tumor aggressiveness and chemoresistance [10,17,18,19,20,21,22]. Our group earlier reported that PCa cells accumulate lipids under hypoxia, which is associated with increased HIF-1α, ATP-citrate lyase, and fatty acid synthase expression [19]. After reoxygenation, PCa cells utilized the stored lipids to proliferate, and more importantly inhibition of CPT1A by etomoxir and stable knockdown of this protein compromised PCa cell growth [19]. Furthermore, the utilization of etomoxir in combination with radiation effectively reduced hypoxia and inhibited cancer cell growth [10]. However, the underlying molecular mechanism of how CPT1A confers PCa tumor aggressiveness, particularly under hypoxic conditions, is still to be determined. To answer this question, we utilized PCa cells with stable knockdown or overexpression of CPT1A and studied the effects of modulating the expression of this protein under hypoxic conditions. We hypothesized that CPT1A expression helps cancer cells to endure hypoxic stress. The results from cell culture and animal studies support our hypothesis and clearly show that CPT1A confers an advantage in PCa cell survival under conditions of chronic hypoxic stress and that knockdown of this protein sensitizes these cells to hypoxia.

## 2. Materials and Methods

### 2.1. Cell Culture and Lentiviral Transfections

Human PCa 22Rv1 and MDA-PCa-2B cells were purchased from the American Type Culture Collection (ATCC, Manassas, Virginia, USA) (Cat# CRL-2505, Cat# CRL-2422) and cultured following the supplier’s instructions. To promote MDA-PCa-2b cell attachment, cell culture plates and dishes were pre-coated with poly-l-lysine (50 µg/mL). Cells were maintained in a humidified incubator with 5% CO_2_ at 37 °C. For this study, we selected 22Rv1 cells, as these cells are known to form xenografts in nude mice [23]. One of the goals of this study was to study the role of CPT1A in PCa cells in vivo, especially under hypoxic microenvironment. We also included MDA PCa 2b cells, a bone metastatic PCa cell line derived from African American patients, to validate a few of the findings. 

The 22Rv1 cells with stable knockdown or overexpression were generated and characterized as reported by Joshi et al. [22]. MDA-PCa-2B cells were transfected with lentiviral particles for shRNA specific for CPT1A prepared at the Functional Genomics Facility at the University of Colorado Anschutz Medical Campus. The Sigma shRNA library utilized was TRCN0000036279 (CPT1A-KD) and the control shRNA (NTshRNA) was SHC202. For CPT1A overexpression (CPT1A-OE), we used the ccsbBroad304-00359 clone from the CCSB-Broad lentiviral library. The 22Rv1 cells and MDA-PCa-2b cells with stable knockdown of CPT1A and their corresponding vectors controls were grown in complete media supplemented with 1 µg/mL puromycin for selection. The 22Rv1 cells with stable overexpression and corresponding empty vector control cells were grown in complete media and selected with 5 µg/mL blasticidin.

### 2.2. Hypoxia Exposure

After cultured cells were attached to the plate as a monolayer, media was replaced with fresh media without disturbing the cells. Then, cells were exposed to normoxia (~21% O_2_) or hypoxia (1% O_2_) for a defined period for each experiment. A BioSpherix X3 Xvivo system (BioSpherix, Parish, NY, USA) was utilized to maintain a hypoxic environment. 

### 2.3. Western Blot Analysis 

Western blotting was performed as described previously [24]. Briefly, an equal amount of protein in whole-cell lysates was separated using Novex Tris-Glycine gels (Invitrogen, ThermoFisher Scientific, Waltham, MA, USA) with varying percentages, and the fractionated proteins were transferred into nitrocellulose membranes (Cat# 1620112, Bio-Rad, Hercules, CA, USA). Membranes were blocked in 5% dry milk prepared in TBS-0.1% Tween buffer and probed individually overnight at 4 °C with each corresponding primary antibody. After washing, membranes were incubated with complementary secondary antibodies for an hour at room temperature. To detect protein band signals, we utilized enhanced chemiluminescence (ECL) following the addition of equal amounts of each substrate agent Clarity Western ECL Substrate (Cat# #170506, Bio-Rad, Hercules, CA, USA). The CPT1A primary antibody was utilized at a 1:1000 dilution and obtained from Proteintech (Cat# 15184-1-AP, Rosemont, IL, USA) or Abcam (Cat#128568, Abcam, Waltham, MA, USA). The β-actin primary antibody was utilized as a loading control at a 1:1000 dilution and was obtained from Abcam (Cat# ab16667, Abcam, Waltham, MA, USA). The α-tubulin was utilized as a loading control at a 1:1000 dilution and was obtained from Abcam (Cat# ab7291, Abcam, Waltham, MA, USA). 

### 2.4. MTT Assay

Cell proliferation was assessed utilizing the 3-(4,5-dimethylthiazol-2-yl)-2,5-diphenyltetrazolium bromide (MTT) reagent (Cat# M5655-100MG, Sigma-Aldrich, St. Louis, MO, USA). Briefly, 2 × 10^3^ cells/well of each cell line were plated in 100 µL of media/well in 96 well plates. Cells were exposed to normoxia or hypoxia for 24, 48, or 72 h. Towards the end, cells were incubated with 20 µL/well MTT reagent (5 mg/mL) for 2 hours at 37 °C. After incubation, the medium or reagent was carefully removed, leaving attached cells undisturbed, then formazan crystals were dissolved utilizing 200 µL of dimethyl sulfoxide (DMSO) and incubated in the dark for 20 min. Absorbance was determined at 560 nm and 650 nm utilizing a Molecular Devices precision microplate reader.

### 2.5. Trypan Blue Exclusion Assay

Trypan blue exclusion assay was utilized to assess the number of cells (live and dead). At the end of the experiments, cells were collected and a 1:1 solution of cell suspension and trypan blue 0.4% solution (Cat# 15250061, Gibco, ThermoFisher Scientific, Waltham, MA, USA) was prepared and then counted under a hemocytometer.

### 2.6. Colony Formation Assay

Cells were cultured at a density of 1 × 10^3^ per well in 6-well plates in triplicate. Cells were maintained in a normoxic or hypoxic environment for the duration of the experiment. Colonies were determined to be at least ≥50 cells/colony and counted at day 7. Additional growth (up to 10 days) was permitted so that colonies were visible. Media were removed and colonies were washed with 1× PBS and fixed using a 3:1 methanol/acetic acid solution for 20 min at room temperature. Colonies were stained with a solution containing 0.5% crystal violet diluted in methanol.

### 2.7. Spheroid Assay

Spheroid culture assay was performed as previously described [10]. Cells were plated at a density of 2500 cells/well in ultra-low attachment six-well culture plates (Cat# 3471, Corning, NY, USA) in DMEM/F12 spheroid media (Cat# 21331020, Gibco, ThermoFisher Scientific, Waltham, MA, USA) and media supplemented with B27 (Cat#17504044, Gibco, ThermoFisher Scientific, Waltham, MA, USA) and N-2 (Cat# 17502001, Gibco, ThermoFisher Scientific, Waltham, MA, USA). Spheres were counted on day 7 and collected on day 10. Images were captured using a PrimoVert Zeiss light microscope (Carl Zeiss Microscopy, White Plains, NY, USA). The experiment was performed in triplicate and the sphere numbers were counted. Images were analyzed to assess the sphere area using Zeiss ZEN Blue lite software (Carl Zeiss Microscopy, White Plains, NY, USA) (10 spheres per group, when possible; if the number was lower, all spheres were measured). 

### 2.8. Histopathology

The formalin-fixed and paraffin-embedded tissues were cut into 5 μm thick sections on glass slides and stained with hematoxylin and eosin (H&E) for pathological evaluation. The area of necrotic tissue present in the tissue section was measured using ImageJ software (version 1.53e). The percentage of the necrotic area was calculated from the total area of the tissue section.

### 2.9. Immunohistochemistry (IHC)

IHC was performed on formalin-fixed, paraffin-embedded tissue sections of xenograft tumors. Briefly, slides were deparaffinized and re-hydrated utilizing xylene, 100% ETOH, 70% ETOH, and DI H_2_O. Antigen retrieval was performed using 10 nM of sodium citrate solution (pH 6.0) at sub-boiling temperature for 1 h. Slides were washed in PBS-Tween 0.2% before utilizing BLOXALL Blocking solution (cat# SP-6000-100, Vector Laboratories, Burlingame, CA, USA). Then, after washing in PBS-Tween 0.2%, slides were blocked in PBS-5% BSA for 1 h at 4 °C. After overnight incubation with each primary antibody, slides were washed in PBS-Tween 0.2% and incubated with ImmPRESS^®^-AP Horse Anti-Rabbit IgG Polymer Detection Kit, Alkaline Phosphatase (cat# MP-5401, Vector laboratories, Burlingame, CA, USA), or ImmPRESS^®^-AP Horse Anti-Mouse IgG Polymer Detection Kit, Alkaline Phosphatase (cat# MP-5402, Vector laboratories, Burlingame, CA, USA). For substrate development, we utilized ImmPACT Vector Red (cat# SK-5105, Vector laboratories, Burlingame, CA, USA) prepared according to the manufacturer’s instructions. Slides were washed twice in PBS-Tween 0.2% and then counterstained with hematoxylin. Slides were submerged in ETOH with 1% HCl solution and then in water to remove the extra dye. Finally, slides were washed consequently in DI H_2_O, 70% ETOH, 95% ETOH, 100% ETOH, and xylene. Cells were mounted utilizing Cytoseal-60 mounting solution and covered with a coverslip. Slides were scanned by NanoZoomer (Hamamatsu, Japan) using 40× lens.

The immunostaining for Ki67 and cyclin D1 was evaluated by counting positive nuclei in the ten representative high-power fields (hpf). The proportion of positive cells to the total number of cells in each field was calculated to be expressed as a percentage. The scoring was conducted manually and few also by digital image analysis using Visiopharm (Visiopharm A/S, Westminster, CO, USA) [24]. The VEGF immunostaining was scored on the basis of the staining intensity in the cytoplasm of cells. Areas with strong staining intensity were scored as ‘+3′, with moderate intensity as ‘+2′, and with mild intensity as ‘+1′. CD31 and LYVE1 immunostaining was evaluated by counting the numbers of the CD31- and LYVE1-positive vessels in the 10 hpf. Antibodies utilized were rabbit anti-Ki67 (1:100 dilution; cat# ab16667, Abcam, Waltham, MA, USA), rabbit anti-cyclin D1 (1:100 dilution, cat# Ab16663, Abcam, Waltham, MA, USA), rabbit anti-CD31 (1:50 dilution; cat# Ab28364, Abcam, Waltham, MA, USA),mouse anti-VEGF (1:100 dilution; cat# ab1316, Abcam, Waltham, MA, USA), rabbit anti-VEGF D (1:100 dilution: cat# Ab15288, Abcam, Waltham, MA, USA), and rabbit anti-LYVE1 (1:100 dilution; cat#ab33682, Abcam, Waltham, MA, USA).

### 2.10. Xenograft Mouse Experiments

Animal care procedures were performed in accordance with the protocol approved by the Institutional Animal Care and Use Committee at Wake Forest University Health Sciences (Winston-Salem, NC, USA). Male athymic nude mice (nu/nu) were purchased from Envigo (Envigo, Indianapolis, IN, USA) at 4–6 weeks of age and were given ad libitum food and water on a 12 h light/dark cycle. For xenograft generation, PCa cells were collected on the day of injection and resuspended in serum-free media. An equal volume of Matrigel Matrix (cat# 354248, Corning, NY, USA) was added to the cells and kept on ice. Mice were anesthetized and 1.1 × 10^6^ cells were injected subcutaneously on both flanks of each mouse. Tumor growth was assessed with calipers and volume was calculated as Width (mm)^2^ × Length (mm) × 0.52 = Volume (mm^3^).

### 2.11. Immunofluorescence 

Mice were injected pimonidazole (120 mg/kg, IP) (cat# HP1-100Kit, Hypoxyprobe™-1, Hypoxyprobe, Burlington, MA, USA) two hours before sacrificing. A portion of the tumor was immediately stored in OCT. Slides were fixed in 4% paraformaldehyde in PBS. Permeabilization was performed with 1× PBS-0.25% Triton ×-100 for 10 min. Then, slides were washed three times in 1× PBS and blocked with 1% BSA for 1 h at room temperature. Appropriate primary antibody was diluted in 1% BSA in PBS and incubated overnight at 4 °C. The primary antibodies utilized were rabbit anti-Ki67 (Abcam, cat# ab16667, Abcam, Waltham, MA, USA) used at a 1:200 dilution and rabbit anti-cyclin D1 (cat# ab16663, used at a 1:200 dilution, Abcam, Waltham, MA, USA). After primary antibody incubation, slides were washed three times for 5 min in 1× PBS. For secondary antibody incubation, we utilized Alexa Fluor ^®^ 555 Anti-Rabbit IgG Fab2 molecular probe (Cat# #4413S, Cell Signaling Technologies, Danvers, MA, USA). For Hypoxyprobe, we utilized FITC-conjugated monoclonal antibody against pimonidazole. Slides were then mounted using a mounting medium containing DAPI (cat# H-1200-10, Vector Laboratories, Burlingame, CA, USA). Slides were visualized using an oil immersion 40x objective in a Zeiss LSM 800 confocal microscope (Carl Zeiss Microscopy, White Plains, NY, USA). The fluorescent intensity of the images was measured using ImageJ (version 1.53e) by analyzing 10 images for each condition. For every image, 5 random areas were measured for fluorescent intensity. An average of 5 measurements was used as the representative fluorescence of each image. Colocalization of Ki67/cyclin D1 and pimonidazole was measured via Pearson’s correlation using ImageJ Colol2 plugin.

### 2.12. TCGA (The Cancer Genome Atlas) Data Analysis

We downloaded preprocessed gene expression data [log_2_(x + 1)-transformed with RPKM] for The Cancer Genome Atlas Prostate Adenocarcinoma (TCGA-PRAD) RNA-seq gene expression dataset from the cBioPortal database [25,26]. The correlation between CPT1A and HIF1A was evaluated using Pearson’s correlation test.

### 2.13. Statistical Analysis 

Statistical analysis was performed utilizing the SigmaStat 4.0 software (SigmaStat, San Jose, CA, USA) and GraphPad Prism 9 (GraphPad Software, San Diego, CA, USA). To evaluate differences between experimental variables, we utilized *t*-test or one-way or two-way ANOVAs with Tukey’s multiple comparisons test. Differences were considered statistically significant at *p* values equal to or below 0.05 (*p* ≤ 0.05).

## 3. Results

### 3.1. Role of CPT1A in Regulating PCa Cell Proliferation and Colony Formation under Hypoxic Conditions

**Figure 1:**
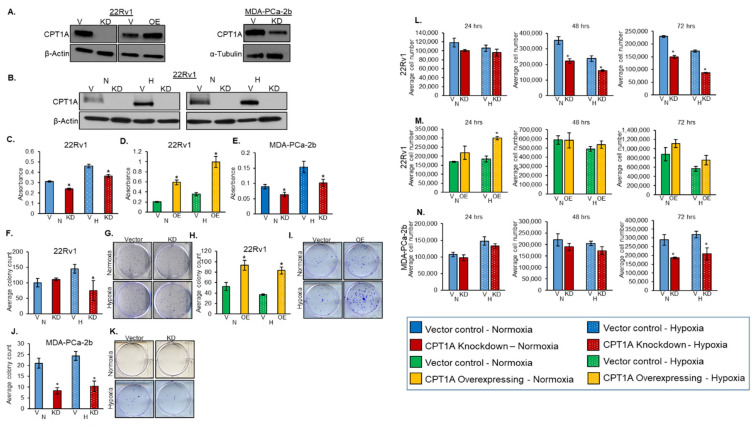
Role of CPT1A in regulating the PCa cell survival, proliferation, and colony formation under hypoxic conditions. (**A**) CPT1A expression was assessed in PCa cells with stable knockdown (KD) or overexpression (OE) of this protein compared to respective vector control cells (V) by Western blotting. The membranes were stripped and re-probed for β-actin or α-tubulin. CPT1A expression was assessed in 22Rv1 cells with stable knockdown grown under 6 and 48 h of hypoxic conditions (**B**). Cells were grown under normoxic (~21% O_2_ solid bars, N) or hypoxic (1% O_2_, dotted bars, H) conditions for 48 h, and cell survival was assessed by MTT assay in 22Rv1 cells (**C**,**D**) (* *p* < 0.001) and MDA-PCa-2b cells (**E**) (* *p* < 0.02) cells with stable knockdown (KD, red) or overexpression (OE, yellow) of CPT1A compared to their corresponding vector controls (V, blue, vector control for CPT1A knockdown cells) (V, green, vector control for CPT1A overexpressing cells). Colony number was assessed in 22Rv1 cells with stable knockdown of CPT1A compared to vector control cells under normoxia or hypoxia. (**F**,**G**) Colony number was assessed in 22Rv1 cells with stable overexpression of CPT1A (OE) compared to vector control cells grown under normoxia or hypoxia (normoxia * *p* < 0.015 and hypoxia * *p* < 0.007). (**H**,**I**) Colony number was assessed in MDA-PCa-2b cells with stable knockdown of CPT1A (KD) compared to vector control cells grown under normoxic or hypoxic conditions (normoxia * *p* < 0.011 and hypoxia * *p* < 0.006). (**J**,**K**) Average cell number (total cell count) was determined under normoxic or hypoxic conditions for 24, 48, and 72 h using trypan blue exclusion assay in 22Rv1 cells with stable knockdown of CPT1A (KD) compared to vector control cells (48 h * *p* < 0.003, * *p* < 0.002; 72 h * *p* < 0.01) (**L**), stable overexpression of CPT1A (OE) compared to vector control cells (* *p* < 0.002) (**M**), and MDA-PCa-2b with stable knockdown of CPT1A (KD) compared to vector control cells (**N**). Results are representative of average ± SEM. Experiments were performed in triplicate. Figure S1: Full blots for Figure 1A- left panel are shown in Figure S1 and full blots for Figure 1A- right panel are shown in Figure S2.

To assess the effects of CPT1A KD or OE on PCa cell proliferation and colony formation, we first confirmed the knockdown and overexpression of stably transfected 22Rv1 cells and knockdown of stably transfected MDA-PCa-2b cells compared to their respective vector controls (Figure 1A, full blot shown in Figures S1 and S2). We also confirmed the CPT1A expression under 6 and 48 h of hypoxic conditions (Figure 1B, full blot shown in Figure S3); both confirmed a strong knockdown of CPT1A expression. We then assessed the effects of CPT1A knockdown (KD) or overexpression (OE) on 22Rv1 PCa cell viability under normoxic and hypoxic conditions. The MTT assay results showed that 22Rv1 PCa cells with CPT1A KD had a statistically significant decrease in viability under both normoxia and hypoxia compared to vector control cells (Figure 1C). Interestingly, we observed a strong increase in cell viability with CPT1A OE under both normoxia and hypoxia compared to vector control cells (Figure 1D). Similar to 22Rv1 cells, we observed a decrease in the viability of CPT1A KD MDA-PCa-2b cells under both normoxia and hypoxia compared to vector control cells (Figure 1E). 

Next, we assessed the role of CPT1A in colony formation by PCa cells under normoxia and hypoxia (Figure 1F–K). We did not observe any differences in colony number in 22Rv1 CPT1A KD cells under normoxia but observed a 48.5% decrease in colony number when these cells were grown under hypoxia (Figure 1F,G). The differences observed in the viability and colony formation potential in the 22Rv1 CPT1A KD cells under normoxic conditions could be explained by the nature of the assays, since MTT is a colorimetric assay that measures metabolic activity based on the reduction of MTT in the cells [27]. In addition, although the difference in colony number was not significant, we did observe a reduction in colony size in 22Rv1 CPT1A KD cells under both conditions. The colony formation was enhanced in 22Rv1 cells with CPT1A OE under both normoxia and hypoxia (Figure 1H,I). Furthermore, we assessed colony formation in MDA-PCa-2b cells with CPT1A KD grown under both normoxia and hypoxia (Figure 1J,K). We observed a significant decrease in colony number in cells with stable CPT1A KD in both normoxia (60% decrease; *p* < 0.011) and hypoxia (57% decrease; *p* < 0.006) compared to their respective vector control cells.

Finally, we assessed cell proliferation utilizing the trypan blue exclusion assay. Minimal cell death was observed (results not shown), indicating that any change observed in cell number was not due to excess cell death, and since cell death was minimal, we decided to present the total cell count. We observed a time-dependent decrease in cell proliferation of 22Rv1 cells with CPT1A KD grown under both normoxia and hypoxia (Figure 1L). In general, we observed an increase in 22Rv1 cell proliferation with CPTIA OE, but a statistically significant increase was only observed following 24 h culture under hypoxia (*p* < 0.003) (Figure 1M). Finally, we observed a decrease in proliferation of MDA-PCa-2b cells with CPT1A KD, but a statistically significant decrease was observed only after 72 h culture under hypoxia (34.4% decrease; *p* < 0.05) (Figure 1N). Together, these results suggest that CPT1A regulates the viability, proliferation, and clonogenicity of PCa cells under both normoxic and hypoxic conditions.

### 3.2. Role of CPT1A in Regulating Sphere Formation by PCa Cells under Hypoxic Condition

**Figure 2:**
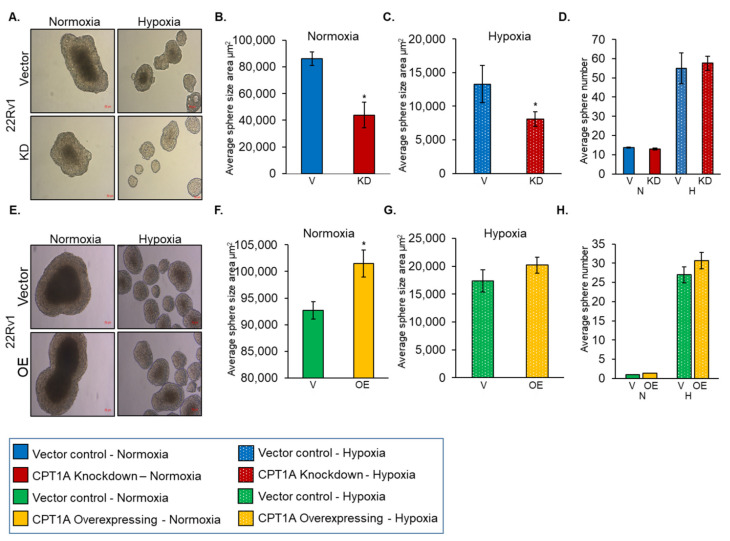
Role of CPT1A expression in 22Rv1 sphere formation. Sphere size and number were assessed in 22Rv1 with stable knockdown (KD, red) compared to vector control (V, blue) (* *p* < 0.001; *n* = 3, area calculated for *n* = 8 spheres) (**A**–**D**) or overexpression (OE, yellow) compared to vector control (V, green) (* *p* < 0.04; experiments performed in triplicate, area calculated for *n* = 5) (**E**–**H**) grown under normoxic (~21% O2, solid bars, N) or hypoxic (1% O2, dotted bars, H) conditions for 10 days. Images were captured under an inverted light microscope and representative images are shown at 20× magnification. Data are representative of average ± SEM. Scale bar = 50 µm.

The 22Rv1 cells with stable KD or OE of CPT1A were cultured as spheres under both normoxia and hypoxia (Figure 2). Earlier, we have effectively utilized this model to characterize the effects of CPT1A inhibition and radiation on hypoxic areas [10]. In 22Rv1 cells with stable CPT1A KD, we observed a significant decrease in the sphere area under both normoxic (Figure 2A,B) and hypoxic conditions (Figure 2A,C) compared to vector control cells. Interestingly, we observed that 22Rv1 cells with CPT1A KD lacked a dense core, especially under hypoxia (Figure 2A). This could be due to the limitation in size of these spheres, since they are not able to grow beyond a certain size in situations of extreme stress such as hypoxia. Interestingly, we observed that 22Rv1 cells with vector control and stable CPT1A KD showed significant increases in the numbers of spheres under hypoxia, but these were significantly smaller than spheres formed under normoxic conditions (Figure 2A,D). 

We then grew 22Rv1 cells with stable overexpression of CPT1A under both normoxia and hypoxia and assessed the sphere size and number. We observed that spheres formed under normoxic conditions were bigger than spheres formed under hypoxia (Figure 2E). The 22Rv1 cells with CPT1A OE produced spheres significantly bigger in size under normoxia (*p* < 0.04) (Figure 2E,F). CPT1A OE 22Rv1 cells grown under hypoxia produced bigger spheres, but the difference was not statistically significant (Figure 2E,G). Interestingly, both vector control and CPT1A OE 22Rv1 cells grown in low oxygen conditions produced a higher number of spheres compared to cells grown under normoxia (Figure 2H).

### 3.3. The Effect of CPT1A Expression on In Vivo Growth of 22Rv1 Cells in Immunocompromised Athymic Nude Mice

**Figure 3:**
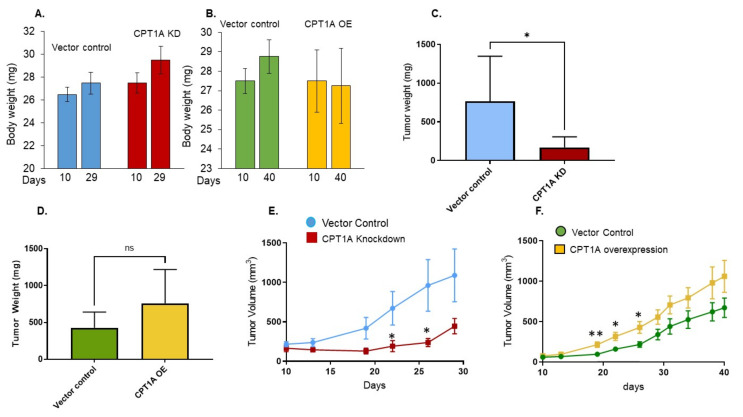
The effects of CPT1A expression on in vivo growth of 22Rv1 cells in immunocompromised athymic nude mice. (**A**) Mice were injected with 22Rv1 cells with vector control (V, blue) or stable knockdown (KD, red) and body weight (g) was documented at 10 and 29 days after cell injection. (**B**) Mice were injected with 22Rv1 cells with vector control (V, green) or stable overexpression (OE, yellow) and body weight (g) was documented at 10 and 40 days after injection. (**C**,**D**) Tumor weight (mg) was assessed at the end of the experiment: day 29 (CPT1A knockdown) (vector control n = 6; CPT1A KD *n* = 7) and day 40 (CPT1A overexpression) (vector control *n* = 8; CPT1 OE n = 8) (* *p* < 0.05). (**E**) On each mentioned day, tumor volume (mm3) was assessed for visible tumors from 22Rv1 CPT1A knockdown cells (red) compared to vector control (blue) and presented as mean ± SEM (* *p* < 0.05). (**F**) On each mentioned day, tumor volume (mm3) was assessed in tumors from 22Rv1 CPT1A overexpressing cells (yellow) compared to vector control (green) and presented as mean ± SEM (*n* = 8 each group) (* *p* < 0.05; ** *p* < 0.01). ns = not significant.

To assess the effects of CPT1A KD or OE on tumor development in an immunocompromised mouse model, we injected these 22Rv1 cells with stable CPT1A KD or OE as well as respective vector control cells in male nude mice and monitored their growth. Throughout, we did not observe any significant differences in body weight in either group of animals (Figure 3A,B). We observed a significant decrease in tumor weight in animals injected with CPT1A KD 22Rv1 cells compared to vector control cells (*p* < 0.05) (Figure 3C). Interestingly, we also observed an increase in tumor weight in animals injected with 22Rv1 CPT1A OE cells, although this difference was not statistically significant (Figure 3D). Our results also showed that mice injected with 22Rv1 cells with CPT1A KD throughout exhibited slower tumor growth compared to mice injected with vector control cells (Figure 3E). On the other hand, animals injected with 22Rv1 cells with CPT1A OE showed an increase in tumor volume compared to animals injected with vector control cells (Figure 3F). Noticeably, due to variations in tumor size, these results were not statistically significant after day 25. However, tumor volume was consistently elevated in the CPT1A OE group. Overall, these results suggest that the status of CPT1A expression can affect 22Rv1 tumor development, as evidenced by the limited growth in tumors with CPT1A KD and enhanced growth in tumors with CPT1A OE.

### 3.4. CPT1A Expression Regulates the Extent of Hypoxic Regions and Expression of Proliferation Biomarkers in 22Rv1 Tumors

**Figure 4:**
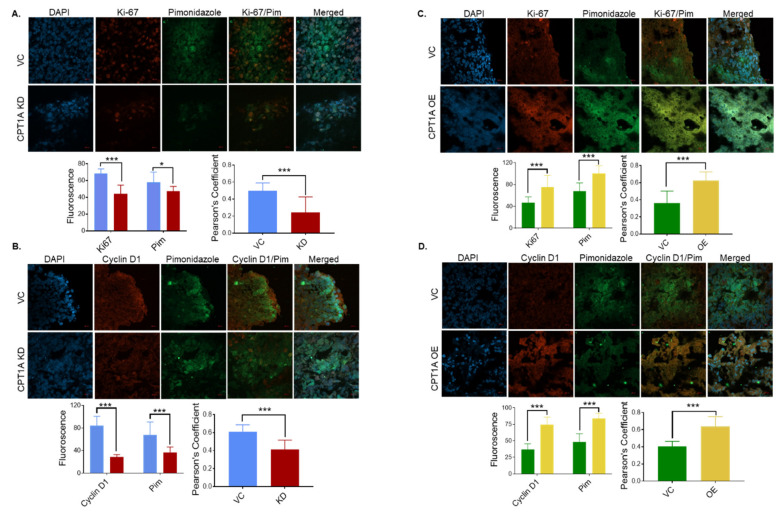
CPT1A regulates cell proliferation in hypoxic regions of 22Rv1 tumors. Mice were injected pimonidazole (120 mg/kg, IP) before sacrifice as described in the Methods, and subsequently tumor tissues were placed in OCT. Next, OCT sections were stained with FITC conjugated antibody anti-pimonidazole (green) and proliferation biomarkers (Ki67 and cyclin D1) using rabbit anti-Ki-67 and rabbit anti-cyclin D1 primary antibodies. Both Ki67 and cyclin D1 were stained using Alexa Fluor 555-tagged secondary antibody (red) and slides were analyzed by confocal microscopy. (**A**–**D**) Representative photomicrographs (at 40×) are shown for vector control (VC) versus CPT1A knockdown (KD) for Ki67 and cyclin D1 (**A**,**B**). Representative photomicrographs (at 40x) are shown for vector control (VC) versus CPT1A overexpressing (OE) for Ki67 and cyclin D1 (**C**,**D**). Images were quantified to measure the relative expression of Ki67, cyclin D1, and pimonidazole. The fluorescent intensity of the images was measured using Image J by analyzing 10 images for each condition and 5 measurements for each image (left graph in each panel). Colocalization of Ki67/cyclin D1 and pimonidazole was measured as Pearson’s correlation by ImagJ (version 1.53e) Colol2 plugin (right graph in each panel). Graphs were plotted with GraphPad Prism. Note: * < *p* = 0.05, *** < *p* = 0.001. Scale bar = 20 µm.

As a tumor grows, limited oxygenation and abnormal microvasculature result in hypoxic conditions [20]. The energetic demands of cancer cells under this harsh environment also need to be satisfied to maintain growth, and CPT1A has emerged as a key player in cancer cell survival since it catalyzes the rate-limiting step for fat oxidation [18,28]. To further assess CPT1A’s role in PCa cell survival under hypoxic conditions, we utilized pimonidazole, a 2-nitroimidazole compound, which forms covalent bonds with cellular macromolecules at oxygen levels below 1.3% [29]. Tumors with CPT1A KD showed significantly lower Ki67 (*p* < 0.001) and pimonidazole+ hypoxic areas (*p* < 0.05) compared to vector control (Figure 4A). There was also a significant decrease in Pearson’s coefficient, a measure of colocalization of Ki67 and pimonidazole in tumors, in the CPT1A KD group compared to vector control (*p* < 0.001) (Figure 4A). Similarly, we observed a decrease in the cyclin D1 level (*p* < 0.001) and pimonidazole+ hypoxic areas (*p* < 0.001) in the CPT1A KD group compared to the vector control (Figure 4B). We also observed a significant decrease in colocalization of cyclin D1 and pimonidazole in the CPT1A KD group compared to the vector control group (*p* < 0.001) (Figure 4B). 

Similarly, we studied whether CPT1A overexpression affected proliferation, particularly in hypoxic areas. We observed significant increases in Ki67 (*p* < 0.001) and pimonidazole (*p* < 0.001) fluorescence in tumors with CPT1A OE cells compared to vector control (Figure 4C). Similarly, there was a significant increase in the Pearson’s coefficient (Ki67 and pimonidazole colocalization) in the CPT1A OE tumors compared to vector controls (*p* < 0.001) (Figure 4C). Finally, we also observed increased fluorescence in both pimonidazole (*p* < 0.001) and cyclin D1 (*p* < 0.001) in the CPT1A OE tumors compared to the vector control group (Figure 4D). We also observed a significant increase in the Pearson’s coefficient (cyclin D1 and pimonidazole colocalization) in the CPT1A OE group compared to the vector tumors (*p* < 0.001) (Figure 4D). These results suggest that CPT1A expression could regulate cancer cell survival and proliferation in hypoxic environments.

### 3.5. Necrosis in 22Rv1 Tumors with Stable Knockdown or Overexpression of CPT1A

**Figure 5:**
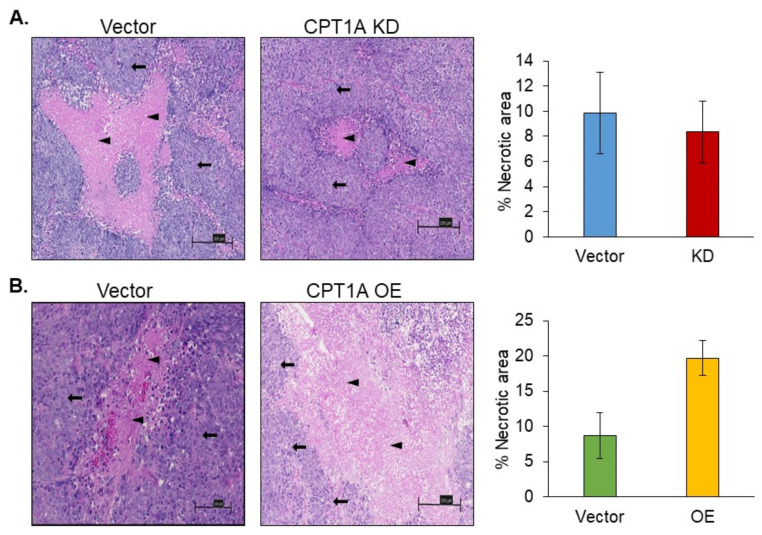
The measure of tumor necrosis in 22Rv1 tumors with stable knockdown or overexpression of CPT1A. Formalin-fixed tissues section were stained (H&E) and eosinophilic necrotic cells (black arrowheads) surrounded by the proliferating neoplastic cells (black arrows) forming a necrotic core at the center were analyzed in various groups. Representative images (left) and quantification of necrotic areas (right) are shown for (**A**) vector control and CPT1A KD and (**B**) and vectors control and CPT1A OE. The following numbers of the section were analyzed per group—vector control (*n* = 6), CPT1A KD (*n* = 7), and vector control (*n* = 8), CPT1A OE (*n* = 8). Sections were analyzed and presented as percentages of the necrotic area mean ± SEM. Representative photomicrographs (at 200× magnification) are shown. Scale bar = 200 µm.

We next assessed the presence of necrotic areas in both the CPT1A KD and OE groups compared to their corresponding vector control (Figure 5). Necrotic regions (black arrowheads) surrounded by the proliferating 22Rv1 cells (black arrows) were present in vector controls, CPT1A KD, and CPT1A OE groups at various locations within the tissue sections. The area of necrosis in the CPT1A KD tumors was less than the corresponding vector control tumors but was not statistically significant (Figure 5A). In the CPT1A OE group, the area of necrosis was relatively higher than in the corresponding controls (Figure 5B). Strikingly, these observations were quite similar to sphere data (Figure 2), where we observed a small or no dense core in CPT1A KD cells under hypoxia but a strong dense core in CPT1A OE cells.

### 3.6. Effects of CPT1A Knockdown on Proliferation-, Angiogenesis-, and Lymphangiogenesis-Related Biomarkers

**Figure 6:**
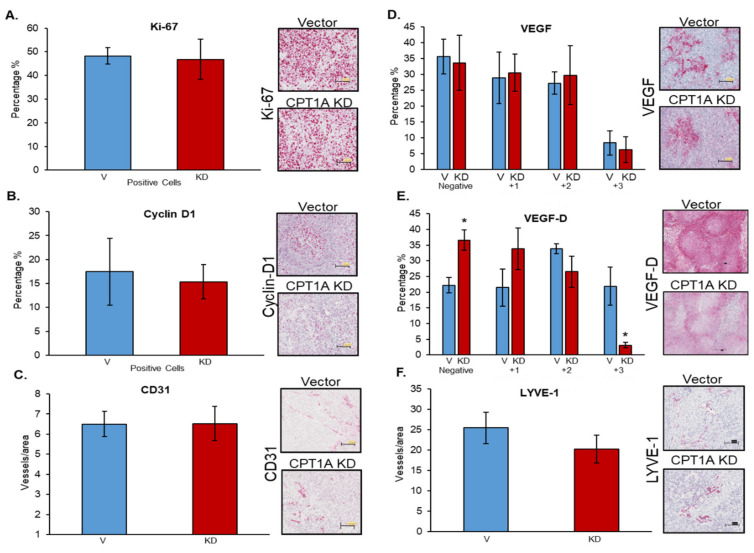
The effects of CPT1A knockdown on proliferation and angiogenesis in 22Rv1 tumors. Tumor tissues from mice bearing vector controls or CPT1A knockdown 22Rv1 tumors were stained to assess various biomarkers by IHC as described in the Methods. Representative images (20× magnification) and quantified data are presented for (**A**) Ki67 (Vector *n* = 6, KD *n* = 7), (**B**) Cyclin D1 (Vector *n* = 3, KD *n* = 5, positive and strong positives grouped for analysis), (**C**) CD31 (Vector *n* = 6, KD *n* = 7), (**D**) VEGF (Vector *n* = 5, KD *n* = 5), (**E**) VEGF-D (Vector *n* = 4, KD *n* = 4) (* *p* < 0.05), and (**F**) LYVE-1 (Vector *n* = 4, KD *n* = 7). IHC data are presented as means ± SEM. Scale bar = 100µm.

Since hypoxia in primary tumors invariably affects angiogenesis and lymphangiogenesis, we next characterized the effects of CPT1A KD on proliferation-, angiogenesis-, and lymphangiogenesis-related biomarkers in tumor tissues. Interestingly, we did not observe any significant difference in the expression of the proliferation marker Ki67 in CPT1A KD tumor tissues compared to vector control (Figure 6A). There was a slight decrease in cyclin D+ cells in the CPT1A KD group compared to the vector control; however, this was not statistically significant due to the high variability in this group (Figure 6B). Next, we explored changes in angiogenesis utilizing CD31, since this marker is expressed in endothelial cells [30]. When assessed, we did not find any differences in microvessel density (CD31 + vessels) between the CPT1A KD and vector control tumors (Figure 6C). We then compared the VEGF expression between these groups, since this growth factor is broadly recognized for its role in angiogenesis and vasculogenesis [30]. We did not observe any significant effects in the percentage of cells with VEGF-positive staining between the CPT1A KD tumors and the vector controls (Figure 6D). The next marker we studied was VEGF-D, an important regulator of lymph vessel growth [31]. We found a significant decrease in the percentage of strongly stained cells (+3) in the CPT1A KD group compared to the vector control (*p* < 0.05). Interestingly, there was also a significant difference in the percentage of VEGF-D-negative cells in the CPT1A KD group (*p* < 0.05) (Figure 6E). Finally, we assessed LYVE-1 microvessel density (LYVE-1+), since this hyaluronan receptor is predominantly expressed in lymphatic vessels [32]. We observed a modest decrease in LYVE-1+ microvessel density in the CPT1A KD tumors compared to the vector group, but this was not statistically significant.

### 3.7. Effects of CPT1A Overexpression on Proliferation-, Angiogenesis-, and Lymphangiogenesis-Related Biomarkers

**Figure 7:**
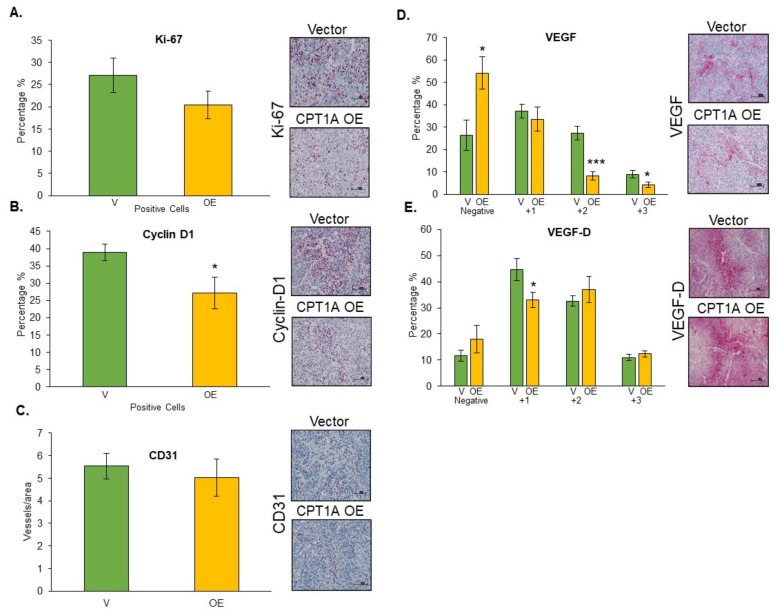
The effect of CPT1A overexpression on proliferation and angiogenesis in 22Rv1 tumors. Tumor tissues from mice bearing vector controls or CPT1A overexpressing 22Rv1 tumors were stained to assess various biomarkers by IHC as described in the Methods. Representative images at 20× magnification and quantified data are presented for (**A**) Ki67 (vector *n* = 8, OE *n* = 8), (**B**) Cyclin D1 (vector *n* = 8, OE *n* = 8) (* *p* < 0.001), (**C**) CD31 (Vector *n* = 8, OE *n* = 8), (**D**) VEGF (Vector *n* = 8, OE *n* = 8) (* *p* < 0.04), and (**E**) VEGF-D (Vector *n* = 8, OE *n* = 7) (* *p* < 0.013, *** *p* < 0.0002, * *p* < 0.04). IHC data are presented as means ± SEM. Scale bar = 100 µm.

We also characterized the effects of CPT1A OE on proliferation-, angiogenesis-, and lymphangiogenesis-related biomarkers in tumor tissues. We did not observe any significant changes, but there was a trend toward lower Ki67 expression in CPT1A OE tumors (Figure 7A), and a slight but statistically significant decrease in cyclin D1 expression was observed (Figure 7B). We did not observe any effect on microvessel density (CD31+ vessels) between the studied groups (Figure 7C). Interestingly, we observed a statistically significant decrease in the percentage of moderately and strongly stained VEGF in the CPT1A OE tumors compared to vector controls, but the same group showed a significant increase in VEGF negative percentage. Similarly, we observed a decrease in VEGF-D expression in CPT1A OE. These results indicate that the observed increase in tumor size when CPT1A is overexpressed is due to differences (such as higher proliferation under hypoxia as shown in Figure 4) that cannot be explained through the utilization of these proliferation and angiogenesis biomarkers.

## 4. Discussion

Recent advances have improved our understanding of the underlying genomic complexity of PCa, changing the therapeutic landscape in the last decade [33]. However, PCa is still one of the most common non-cutaneous malignancies diagnosed in males and the second leading cause of cancer deaths in the United States, making it a formidable healthcare issue to be addressed [1]. Thus, a better understanding of the mechanism/s that both promote and maintain the growth of PCa cells in their harsh microenvironments, such as hypoxia, represents an excellent opportunity to effectively manage this disease. Several studies have shown that hypoxia in primary tumors is associated with PCa progression, high risk of disease recurrence, and treatment failure, including surgery, radiation, and hormone therapy [6,7,8,34,35,36,37,38]. The results from the present study suggest that CPT1A regulates PCa cell survival, clonogenicity, and sphere formation under hypoxic conditions. Moreover, CPT1A regulated the in vivo growth of PCa cells in nude mice. Lastly, CPT1A expression seems to affect PCa cell survival and proliferation in hypoxic areas in tumors.

Rapid tumor growth associated with limited oxygenation and abnormal microvasculature results in a hypoxic microenvironment [20]. Hypoxia promotes a multitude of genetic and epigenetic changes, effectively altering the dynamics of the tumor microenvironment, resulting in increased angiogenesis, glycolysis, stemness, metabolic alterations, and survival of highly aggressive clones [6,39,40]. CPT1A has emerged as a key player in cancer cell survival, since it catalyzes the rate-limiting step for fat oxidation [18,28]. CPT1A expression is increased in various cancers, including prostate, ovarian, glioblastoma, lymphoma, breast, gastric, and colon cancers [18]; however, it is hard to discern CPT1A expression and activity in the hypoxic regions of the tumor using the available clinical samples. The gene expression data of prostate cancer (PRAD) patients in The Cancer Genome Atlas (TCGA) cohort suggested a moderate positive correlation (*r* = 0.22; *p* = 5.7 × 10^−8^) between HIF1A and CPT1A expression (Figure S4). However, more studies are warranted to assess CPT1A protein expression and activity and its colocalization with hypoxic conditions or the expression of hypoxic biomarkers in clinical tumor samples. CPT1A could affect the expression of various genes through epigenetic changes, i.e., through the end-product of β-oxidation (acetyl CoA) [41]. Interestingly, our group recently reported that CPT1A-mediated fat oxidation is able to modify cell growth and therapy via acetylome modification [18,22]. Interestingly, this higher acetylation was associated with less sensitivity of PCa cells to endocrine therapy. Earlier, we reported that the CPT1A inhibitor etomoxir reduced androgen receptor expression (full length and variant form), while CPT1A knockdown increased the sensitivity of PCa cells to androgen and sensitized the cells to anti-androgen therapy [17,18,21]. Lastly, the combination of anti-androgen therapy and CPT1A inhibition showed a robust growth inhibitory effect against PCa [17]. 

Alteration of the lipid metabolism is an important hallmark, since cancer cells must efficiently maintain energy requirements to grow, especially under harsh conditions [42]. Since CPT1A has a major regulatory role in fatty acid oxidation, the absence of this enzyme would leave cells unable to meet the increased energetic demands to grow in hypoxic environments. We previously reported that PCa cells accumulate lipids under hypoxic conditions and then utilize this fat reserve upon reoxygenation to increase their proliferation [19]. In the present study, we observed that CPT1A expression regulated the PCa cell proliferation, colony formation, and sphere size under hypoxic conditions. Interestingly, we observed a decrease in the necrotic core and pimonidazole+ hypoxic areas in the CPT1A KD 22Rv1 tumors, confirming that CPT1A expression might be required in PCa cell survival under hypoxic conditions. It is quite possible that the lesser number of necrotic and pimonidazole+ hypoxic areas are due to the smaller size of CPT1A KD 22Rv1 tumors. As shown in Figure 2, spheres formed by CPT1A KD PCa cells under hypoxia have smaller dense core even when they have a comparable size compared to other groups. Moreover, CPT1A OE tumors exhibited higher percentages of necrotic and pimonidazole+ hypoxic areas with proliferating cells, confirming the role of CPT1A in cell proliferation under these conditions. It is plausible that hypoxic centers could harbor areas of dedifferentiation and selection of malignant cells [43]. Interestingly, other researchers have reported that in a hypoxic environment, hypoxia-induced transcription factors (such as HIF1α) can also stimulate and represses a multitude of genes important for adaptation to the low oxygen environment, including angiogenic inhibitors [44]. In addition, the development of hypoxic regions is not only caused by low vessel density but also vessel quality [45]. We can hypothesize that in this particular subset and experimental conditions, growth was aided by CPT1A status regardless of vascularization. Therefore, even though we did not observe significant changes in proliferation and angiogenesis markers through IHC (Figure 6 and Figure 7), CPT1A expression could regulate the maintenance of malignant hypoxic niches, thereby promoting tumor growth. 

Earlier published studies [18,20] have indicated that targeting CPT1A could potentially be incorporated as part of a therapeutic regimen to curb the rapid growth of cancerous cells. For example, etomoxir, an irreversible inhibitor of CPT1A [46], has been demonstrated to have pharmacological effects with the capability to treat conditions such as congestive heart failure, although its clinical effectivity remains to be studied [47]. Interestingly, studies with etomoxir have also revealed that cancer cells do not completely rely on oxidation of lipids and can be reprogrammed to utilize other sources of energy independent of fatty acid oxidation, such as glutamine, demonstrating the incredible plasticity of these cells [48]. However, it may be that the optimal functionality of CPT1A is essential for cells to survive under conditions of added stress, such as hypoxia or drug treatments, particularly in early tumor development [22]. Inhibition of CPT1A by both knockdown or utilization of its inhibitor etomoxir compromised cancer cell growth following reoxygenation [19]. Additionally, treatment of lung adenocarcinoma cell spheres and PCa cell spheres with a combination of radiation and etomoxir significantly reduced hypoxic regions in both cancer cell models [10]. This treatment combination reduced the expression of biomarkers for stemness, proliferation, and β-oxidation in the lung cancer cells [10]. Furthermore, CPT1A knockdown in LNCaP cells reduced the sphere formation potential of these cells under similar conditions [10]. The results from the present study further support the therapeutic utility of targeting CPT1A expression in cancer cells. 

Finally, early diagnosis of PCa is one of the better predictors of a positive prognosis. Hypoxia is one of the early events in the formation of solid tumors, and hypoxia-related signaling endows cancer cells with more malignant, aggressive, and treatment-refractory characteristics [6]. The results from laboratory and clinical studies suggest the potential of hypoxia signaling biomarkers in the development of better prognostic biomarkers and treatment options [11]. One candidate marker widely utilized to identify hypoxic centers is pimonidazole, a 2-nitroimidazole-based compound that binds covalently to macromolecules at low oxygen conditions [29,37,49]. Studies have also shown the utility of pimonidazole to effectively identify hypoxic areas in tumors that promote more aggressive disease [37,38]. We have also effectively utilized this hypoxic marker to identify areas of low oxygen that can harbor more aggressive cells in sphere models [10]. In the present study, we demonstrated that CPT1A expression regulated cell survival and proliferation in hypoxic centers without major changes in the angiogenesis or lymph-angiogenesis biomarkers. This finding could indicate that patients with increased CPT1A expression, especially in hypoxic areas, could be more predisposed to aggressive tumors. This finding could also be useful to identify high-risk PCa patients.

**Figure 8:**
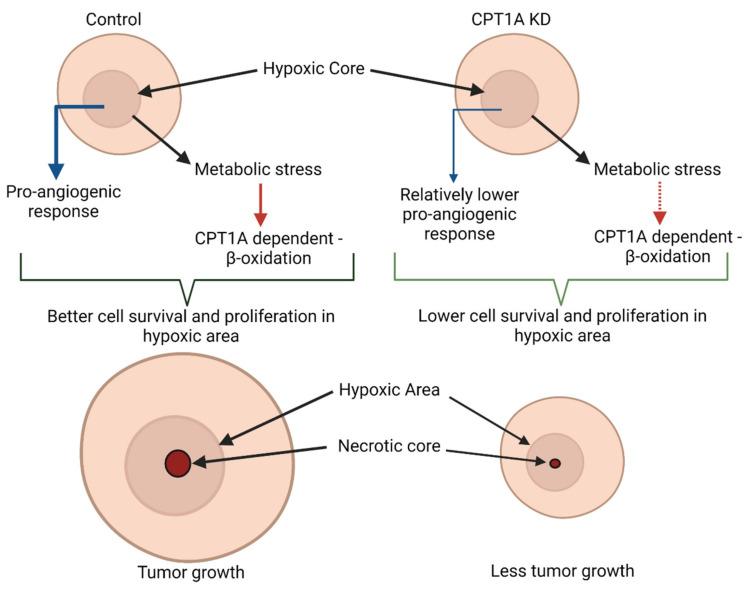
Role of CPT1A expression in regulating PCa cell survival and proliferation in the hypoxic microenvironment. Rapid tumor growth results in a hypoxic microenvironment causing a multitude of genetic and epigenetic changes, effectively altering the dynamics of the tumor microenvironment. In our study, cells with CPT1A overexpression showed enhanced proliferation, stemness, and tumor growth compared to controls under conditions of hypoxia, mildly affecting angiogenic response. The observed proliferative advantage was effectively abrogated with stable CPT1A knockdown.

In summary, the results from the present study highlight a novel role of CPT1A in regulating PCa cell survival and proliferation in the hypoxic microenvironment. We have summarized our results in Figure 8. These outcomes further support CPT1A’s usefulness in the prognosis and therapy of PCa.

## 5. Conclusions

In conclusion, these results show that CPT1A plays a role in PCa cell survival and proliferation under hypoxic conditions. CPT1A expression could influence the extent of hypoxic areas in the tumor as well as PCa cell proliferation in these harsh areas through the maintenance of malignant hypoxic niches. These findings also support the therapeutic utility of targeting CPT1A expression in cancer cells and could potentially be useful to stratify high-risk cancer patients, since patients with increased CPT1A expression could be more predisposed to aggressive disease.

## Data Availability

We downloaded preprocessed gene expression data (log_2_(x + 1)- transformed RPKM) for the TCGA PRAD (TCGA-PRAD) RNA-seq gene expression dataset from the cBioPortal database.

## Supplementary Materials

The following are available online at https://www.mdpi.com/article/10.3390/cancers13246302/s1: Figure S1: Full blots for Figure 1A- left panel. Figure S2: Full blots for Figure 1A- right panel. Figure S3: Full blots for Figure 1B. Figure S4: Correlation between CPT1A and HIF1A expression in the TCGA PRAD cohort.
