# Supplementary material for: Carnitine Palmitoyltransferase 1 Regulates Prostate Cancer Growth under Hypoxia
Source: Cancers (Basel). 2021 Dec 15;13(24):6302. doi: 10.3390/cancers13246302 (PMC8699124; doi:10.3390/cancers13246302)
Supplement: Supplementary file 1 [file cancers-13-06302-s001.zip › cancers-1500059-supplementary.pdf]

Supplementary Materials

Carnitine Palmitoyltransferase 1 Regulates Prostate Cancer Growth under Hypoxia

Leslimar Rios-Colon, Pawan Kumar, Susy Kim, Mitu Sharma, Yixin Su, Ashish Kumar, Sangeeta Singh, Nalexus Stocks, Liang Liu, Molishree Joshi, Isabel R. Schlaepfer, Deepak Kumar and Gagan Deep

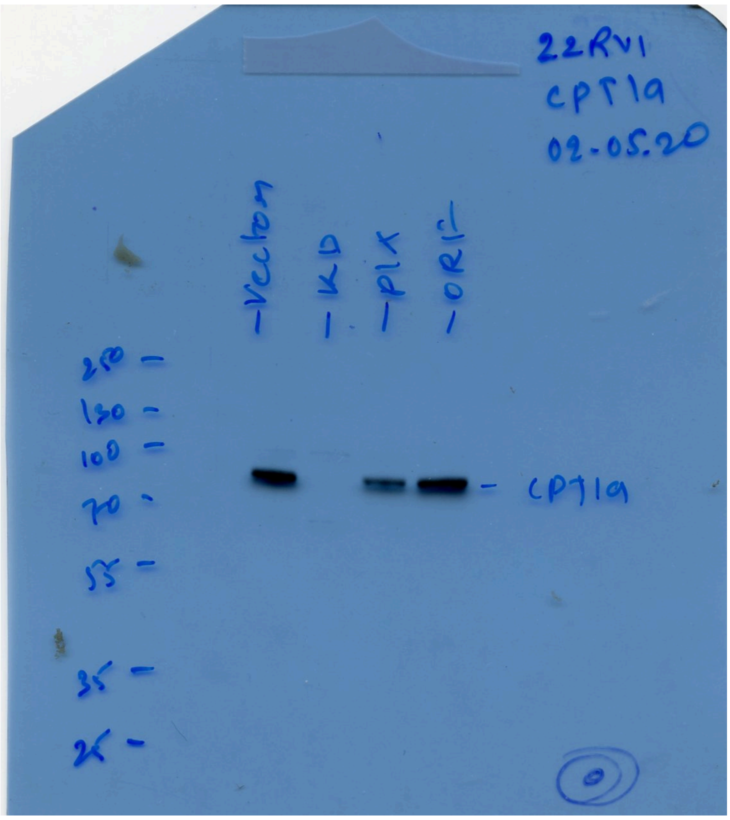

22Rv1 cells(VC,KD, PLX and ORF)- CPT1A
Data shown in Figure 1A, left panel

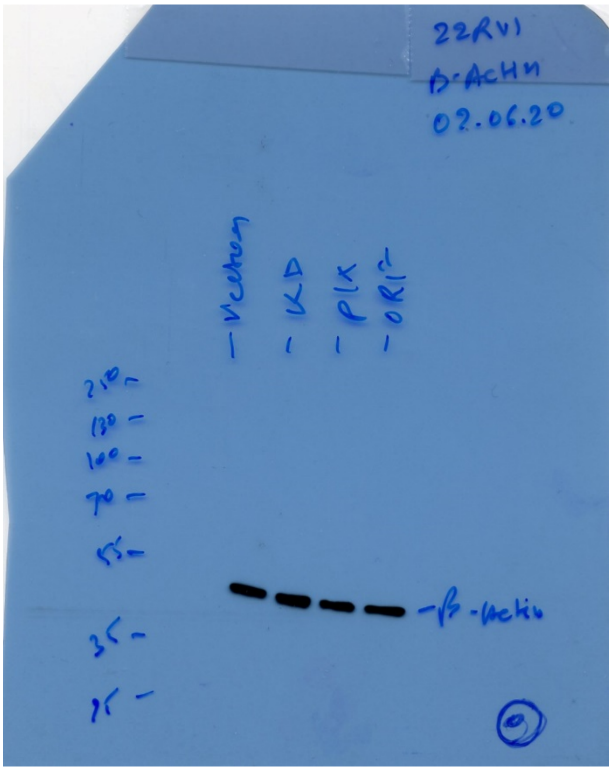

22Rv1 cells(VC,KD, PLX and ORF)- β-actin
Data shown in Figure 1A- left panel

Figure S1: Full blots for Figure 1A- left panel.

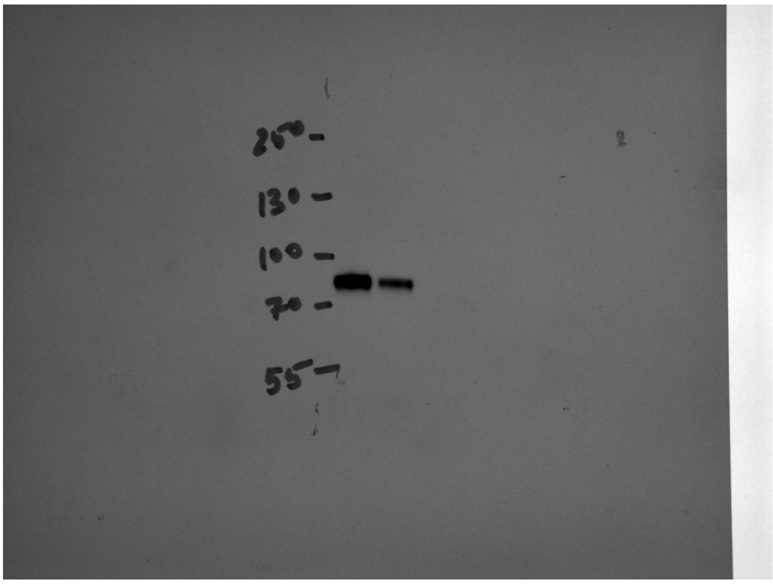

MDA-PCa-2b cells (VC and KD)- CPT1A

Data shown in Figure 1A- right panel

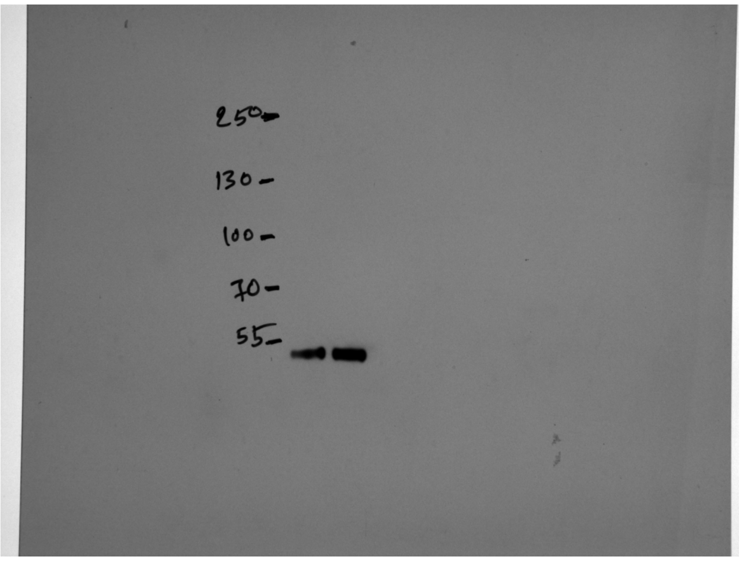

MDA-PCa-2b cells(VC and KD)- α tubulin

Data shown in Figure 1A- right panel

Figure S2. Full blots for Figure 1A- right panel.

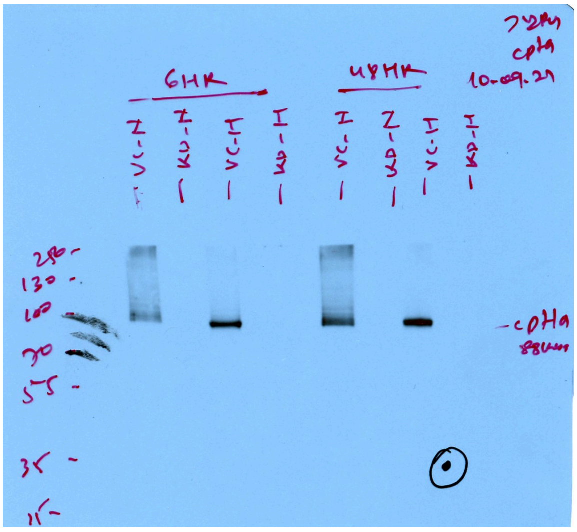

22Rv1 cells(VC and KD)- CPT1A

Data shown in Figure 1B

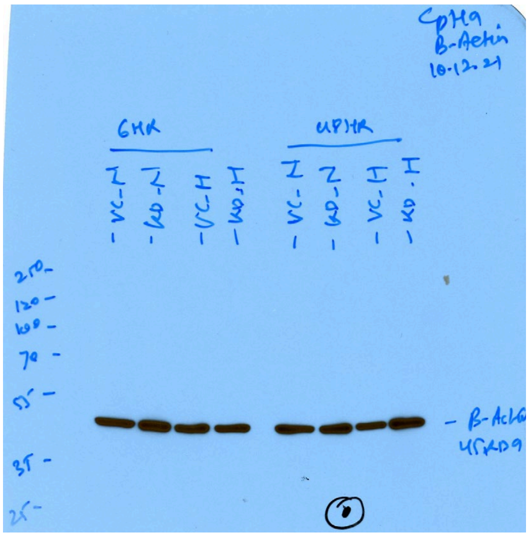

22Rv1 cells(VC and KD)- β-actin

Data shown in Figure 1B

Figure S3. Full blots for Figure 1B.

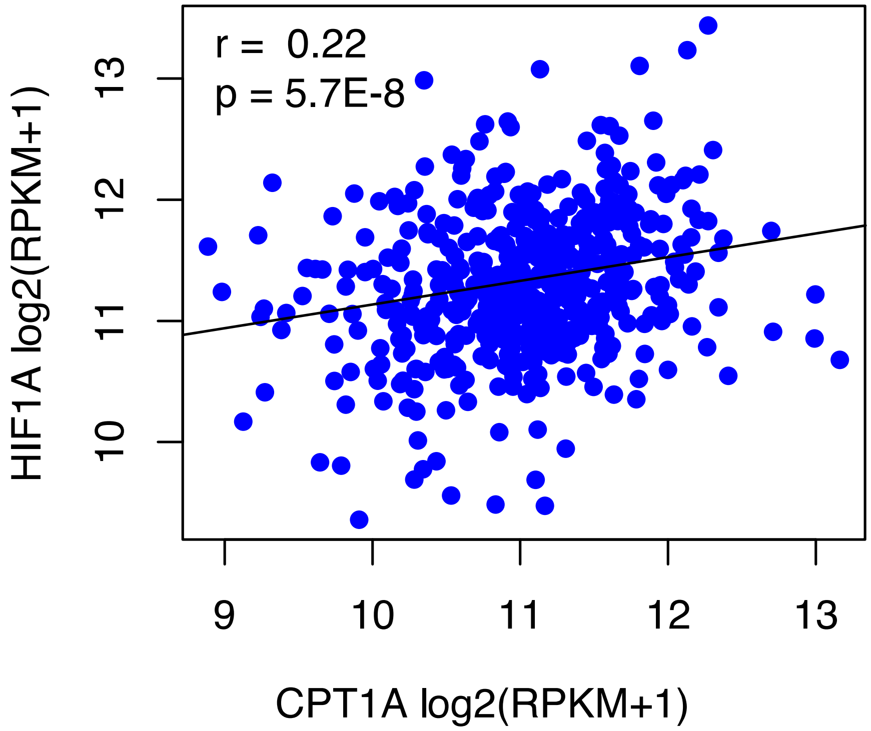

Figure S4. Correlation between CPT1A and HIF1A expression in the TCGA PRAD cohort.
